# Unravelling Novel *SCN5A* Mutations Linked to Brugada Syndrome: Functional, Structural, and Genetic Insights

**DOI:** 10.3390/ijms242015089

**Published:** 2023-10-11

**Authors:** Anthony Frosio, Emanuele Micaglio, Ivan Polsinelli, Serena Calamaio, Dario Melgari, Rachele Prevostini, Andrea Ghiroldi, Anna Binda, Paola Carrera, Marco Villa, Flavio Mastrocinque, Silvia Presi, Raffaele Salerno, Antonio Boccellino, Luigi Anastasia, Giuseppe Ciconte, Stefano Ricagno, Carlo Pappone, Ilaria Rivolta

**Affiliations:** 1 Institute of Molecular and Translational Cardiology (IMTC), IRCCS Policlinico San Donato, 20097 San Donato Milanese, Italy; anthony.frosio@grupposandonato.it (A.F.); emanuele.micaglio@grupposandonato.it (E.M.); ivan.polsinelli@grupposandonato.it (I.P.); serena.calamaio@grupposandonato.it (S.C.); dario.melgari@grupposandonato.it (D.M.); rachele.prevostini@grupposandonato.it (R.P.); andrea.ghiroldi@grupposandonato.it (A.G.); marco.villa@grupposandonato.it (M.V.); anastasia.luigi@hsr.it (L.A.); giuseppe.ciconte@grupposandonato.it (G.C.); stefano.ricagno@unimi.it (S.R.); carlo.pappone@af-ablation.org (C.P.); 2 Arrhythmia and Electrophysiology Department, IRCCS Policlinico San Donato, 20097 San Donato Milanese, Italy; flavio.mastrocinque@grupposandonato.it (F.M.); antonio.boccellino@grupposandonato.it (A.B.); 3 Laboratory of Stem Cells for Tissue Engineering, IRCCS Policlinico San Donato, 20097 San Donato Milanese, Italy; 4 School of Medicine and Surgery, University of Milano-Bicocca, Via Cadore, 48, 20900 Monza, Italy; anna.binda@unimib.it; 5 Laboratory of Clinical Molecular Genetics and Cytogenetics, Unit of Genomics for Diagnosis of Human Diseases, IRCCS San Raffaele Scientific Institute, 20132 Milan, Italy; carrera.paola@hsr.it (P.C.); presi.silvia@hsr.it (S.P.); 6 Faculty of Medicine and Surgery, Vita-Salute San Raffaele University, Via Olgettina, 58, 20132 Milan, Italy; salerno.raffaele@hsr.it; 7 Department of Biosciences, Università degli Studi di Milano, Via Celoria, 26, 20133 Milan, Italy

**Keywords:** Brugada syndrome, sudden cardiac death, arrhythmias, sodium channel, Nav1.5, *SCN5A*, automated patch clamp, mexiletine

## Abstract

Brugada Syndrome (BrS) is a rare inherited cardiac arrhythmia causing potentially fatal ventricular tachycardia or fibrillation, mainly occurring during rest or sleep in young individuals without heart structural issues. It increases the risk of sudden cardiac death, and its characteristic feature is an abnormal ST segment elevation on the ECG. While BrS has diverse genetic origins, a subset of cases can be conducted to mutations in the *SCN5A* gene, which encodes for the Nav1.5 sodium channel. Our study focused on three novel *SCN5A* mutations (p.A344S, p.N347K, and p.D349N) found in unrelated BrS families. Using patch clamp experiments, we found that these mutations disrupted sodium currents: p.A344S reduced current density, while p.N347K and p.D349N completely abolished it, leading to altered voltage dependence and inactivation kinetics when co-expressed with normal channels. We also explored the effects of mexiletine treatment, which can modulate ion channel function. Interestingly, the p.N347K and p.D349N mutations responded well to the treatment, rescuing the current density, while p.A344S showed a limited response. Structural analysis revealed these mutations were positioned in key regions of the channel, impacting its stability and function. This research deepens our understanding of BrS by uncovering the complex relationship between genetic mutations, ion channel behavior, and potential therapeutic interventions.

## 1. Introduction

Brugada syndrome (BrS) is a rare heritable form of cardiac arrhythmia, which often clinically manifests itself with syncope or (resuscitated) cardiac arrest due to either polymorphic ventricular tachycardia (VT) or ventricular fibrillation (VF). Typically, these clinical situations occur at night or at rest in young adults without cardiac structural abnormalities [1]. BrS is characterized by a coved-type ST-segment elevation in the right precordial leads V1 through V3 of the electrocardiogram (ECG) and an increased risk of sudden cardiac death (SCD) [2]. With an estimated variable prevalence of 0–0.1% in Europe/USA and 0–0.94% in Southeast Asia, where it is the leading cause of death in apparently healthy young men, BrS is responsible for 4% of all SCDs and up to 12% of sudden death worldwide in individuals with structurally normal hearts [3,4]. BrS has a complex physiopathology and cannot be defined as a monogenic disease [5,6]. Nevertheless, the sodium voltage-gated channel 5A (*SCN5A*) gene, which encodes the pore-forming α-subunit of the cardiac voltage-gated sodium channel Nav1.5, was the first gene to be associated with BrS in 1998 by Chen and colleagues [7]. Since then, rare variants of more than 20 other genes have been found in BrS patients, but loss-of-function mutations of Nav1.5 still account for 20% to 30% of all genetically diagnosed BrS cases [8,9]. *SCN5A* was also recently assessed as the only undisputed BrS-related gene by the Clinical Genome Resource initiative, while all the other genes were reclassified as disputed [10]. Nav1.5 is the molecular determinant of the inward sodium current (I_Na_) in the heart and underlines the fast depolarization of the cardiac action potential of working myocytes in both the atria and the ventricles [11]. To date, more than 300 Nav1.5 BrS-related mutations have been described, localized throughout the whole protein structure, and associated with a broad spectrum of disease severity [1,12]. Here, we identified and functionally characterized three novel *SCN5A* missense mutations, namely c.1030 G>T (p.A344S), c.1041 C>A (p.N347K), and c.1045 G>A (p.D349N), found in three unrelated BrS patients and their relatives. Given the loss-of-function effect revealed from the patch clamp approach, the effect of chronic exposure to mexiletine was challenged to explore the possibility of a functional rescue. Finally, as all the three mutations are located within the P-loop comprised between the S5 and S6 segments of domain I of the channel, we inquired into their structural significance, shedding lights on these residues’ roles in Nav1.5 and their potential impact on channel function.

## 2. Results

### 2.1. Proband Patient Characteristics

**Figure 1:**
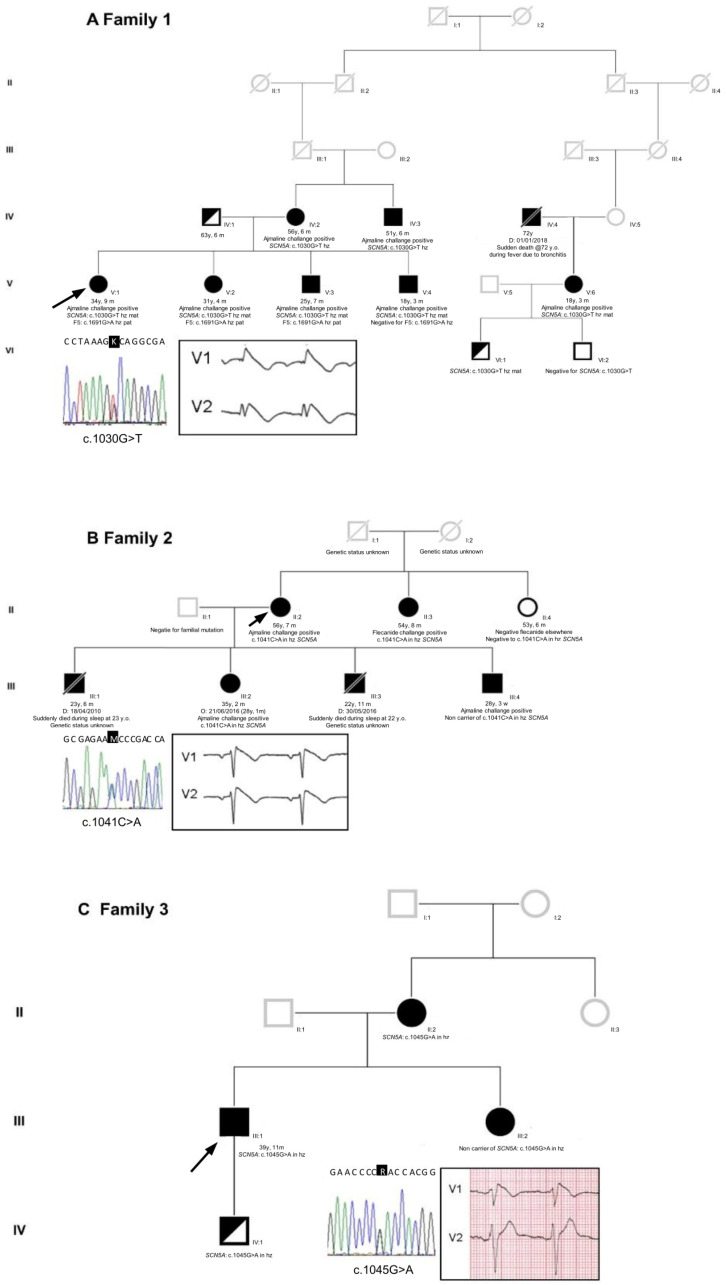
Pedigrees of the three unrelated families. (**A**) Family 1, (**B**) Family 2, and (**C**) Family 3. Arrows indicate the proband in each family. Squares: male; circles: female; deceased individuals are indicated with a slash; BrS individuals are indicated with black filled symbols. Reported are also the proband’s sequence identification of the base substitution and her/his ECG in the insets. Only in the proband of family 1 was the BrS pattern drug-induced; in families 2 and 3, the proband’s ECG type 1 BrS pattern was spontaneous (for complete ECGs, see Figure S1 in the Supplementary Materials).

The family 1 proband was a 34-year-old Caucasian woman, who presented with a family history of BrS (both mother and maternal uncle, see Figure 1 and the Supplementary Materials). She complained of brief episodes of sudden palpitations and occasional syncope during fever. She underwent the ajmaline challenge, which yielded a type 1 BrS ECG result (Figure 1 and Figure S1), while an electrophysiological study (EPS) was negative. Interestingly, prior to the arrhythmologic screening, the proband experienced a stroke episode, with the detection of a heterozygous c.1691G>A variant in the F5 gene (so-called “Leiden Factor V”) with paternal origin and segregating in her family with a thrombophilic phenotype. Further genetic testing revealed the novel heterozygous missense variant NM_198056.2: c.1030G>T (p.A344S) in the *SCN5A* gene [LOVD: https://databases.lovd.nl/shared/individuals/00435247 (accessed on 21 July 2023)] (Figure 1). No other variant was detected except for the F5 Leiden variant, which was identified before the clinical diagnosis of BrS.

The family 2 proband was a 56-year-old Caucasian woman with a family history of sudden cardiac death during night sleep (see Figure 1 and the Supplementary Materials). She had a history of sudden-onset palpitations. The ECG revealed a spontaneous type 1 BrS pattern (Figure 1 and Figure S1). The EPS resulted positive for ventricular fibrillation inducibility, and she subsequently underwent an ICD implant. Genetic testing unveiled a novel heterozygous missense variant, NM_198056.2: c.1041C>A (p.N347K), in the *SCN5A* gene [LOVD: “https://databases.lovd.nl/shared/individuals/00435248 (accessed on 21 July 2023)] (Figure 1).

Lastly, the family 3 proband was a 40-year-old Caucasian man with a family history of cardiac arrhythmias in his mother and maternal aunt (see Figure 1 and the Supplementary Materials). He survived a cardiac arrest at the age of 33 due to spontaneous ventricular arrhythmias. He presented with a spontaneous type 1 BrS ECG pattern (Figure 1 and Figure S1). Therefore, he underwent an ICD implant and received quinidine therapy. Genetic testing revealed a novel heterozygous missense variant, NM_198056.2: c.1045G>A (p.D349N), in the *SCN5A* gene [LOVD: https://databases.lovd.nl/shared/variants/0000295484#00018523 (accessed on 21 July 2023)] (Figure 1).

Sanger sequencing confirmed all the three variants found in the *SCN5A* gene.

### 2.2. Genetic Testing Results and In Silico Prediction

Based on current knowledge, all three heterozygous variants in the *SCN5A* gene are exceptionally rare in the general population, with an estimated frequency of 1 in 248,734 individuals for the c.1030G>T variant; 1 in 248,950 individuals for the c.1045G>A variant, and an unknown frequency for the c.1041C>A variant. All three lack an entry in the GnomAD database, but their locus is covered in the GnomAD genomes, with an average coverage of 33.9 (median coverage of 33) for the c.1030G>T variant, of 33.5 (median coverage of 33) for the c.1041C>A variant, and of 58.2 (median coverage of 53) for the c.1045G>A variant. Furthermore, 95.52%, 94.22%, and 99.81% of the samples had a coverage higher than 20× [source: https://varsome.com/variant/hg19/rs1372770367?annotation-mode=germline (accessed on 21 July 2023)], respectively. To date, neither structural models nor topological predictions for these variants are available.

In terms of the in silico predictions, the three variants were evaluated using nine software tools: CADD, Polyphen2, Mutation Taster, SIFT, EIGEN, FATHMM, REVEL, METARNN, and BayesDel software (https://fenglab.chpc.utah.edu/BayesDel/BayesDel.html, accessed on 21 July 2023). The latter exhibited viable parameters of specificity (87.7%) and sensitivity (90.5%), as observed with the following link: (https://varsome.com/about/resources/germline-implementation/#insilicopredictions accessed on 21 July 2023)). These predictions were generated through germline in silico calibrations based on dbNSFP, utilizing a pathogenicity score ranging from −1.11707 to 0.750927. Higher scores indicated an increased likelihood of pathogenicity, with a proposed a cutoff value of 0.0692655 to distinguish between deleterious (‘D’) and tolerated (‘T’) variants. All three variants were predicted as “pathogenic” in five out of the nine software tools; in particular, BayesDel software was attributed a score of 0.1807, 0.2307, and 0.4573 for the c.1030G>T, c.1041C>A, and c.1045G>A variants, respectively; thus, all of them were classified as “D” (see the Supplementary Materials for the score obtained using the variants with the other indicated software tools and for the allele frequency).

### 2.3. Functional Analysis and Protein Localization

**Figure 2:**
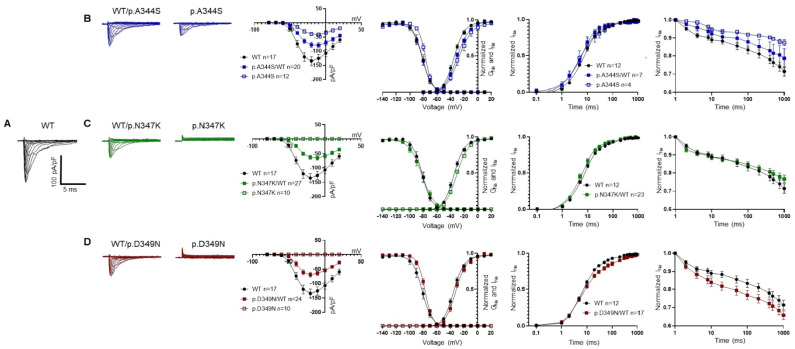
Electrophysiological characterization of Nav1.5 constructs. Representative current traces and their biophysical properties measured via the patch clamp approach in HEK293 cells overexpressing the constructs as indicated: WT in black (**A**), WT/p.A344S or p.A344S in blue (**B**), WT/p.N347K or p.N347K in green (**C**), and WT/p. D349N or p.D349N in red (**D**). Panels B, C, and D present from left to right: families of current traces, current–voltage relationship showing the reduction in the mean current density recorded in the presence of the mutations in heterozygous or homozygous conditions with respect to the WT channel, fitting the data with the Boltzmann equation (see Section 4) provided midpoint activation and inactivation potential reported in Table 1, recovery from inactivation (see Table 1 for the kinetic parameters), and the kinetics of the development of the intermediate inactivation. Data are shown as mean ± SEM.

The electrophysiological properties of the Nav1.5 p.A344S, p.N347K, and p.D349N mutations were addressed in the HEK293 cell line via patch clamp experiments, in whole cell configuration, and compared with the ones of the Nav1.5 WT. Figure 2 and Table 1 condensed the obtained results. Among the three mutations studied, only the p.A344S channel revealed a significant inward current; however, it was affected by a robust reduction when compared with the WT channel. In contrast, the p.N347K and p.D349N mutations completely abolished the I_Na_, when expressed alone. In the experimental conditions that mimicked the genetic background of the patients’, thus forming a 1:1 ratio between the mutated and WT channels, a significant reduction in peak current density was commonly observed. The mutations also impacted the voltage dependence of activation; notably, the presence of p.A344S and p.N347K induced a significant positive shift of 4.5 mV. Additionally, the voltage dependence of inactivation was shifted rightward by 5 mV either by the p.A344S alone or by the presence of p.D349N. The kinetics of fast inactivation were analyzed, only showing a delay in p.A344S alone. While no significant mutation-related differences were observed in recovery from inactivation, the development of an intermediate inactivation process exhibited opposite effects, being accelerated in p.A344S and delayed in p.D349N.

**Figure 3:**
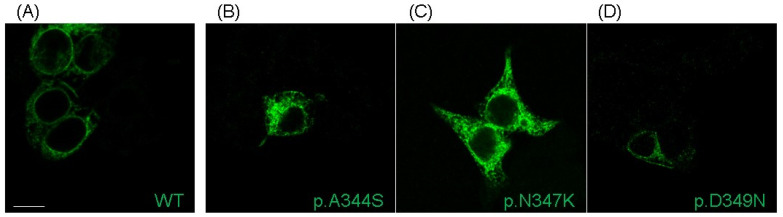
Localization of Nav1.5 WT and mutants transiently transfected in HEK293 cells. Representative immunofluorescence confocal images showing the plasma membrane and cytosolic localization of WT Nav1.5 protein (**A**), along with its p.A344S (**B**), p.N347K (**C**), and p.D349D (**D**) mutants. Nav1.5 is labeled in green. Scale bar: 10 µm.

Immunofluorescence labeling revealed that Nav1.5-mutated proteins were expressed and reached the plasma membrane; similarly to the WT channel, the cytosolic accumulation that was present in the acquired images (and also in the ones regarding the WT channel) was probably due to the overexpression of proteins (Figure 3). Thus, the current reduction observed in all three mutations was not caused by a complete impairment of the trafficking towards the cell membrane.

**Figure 4:**
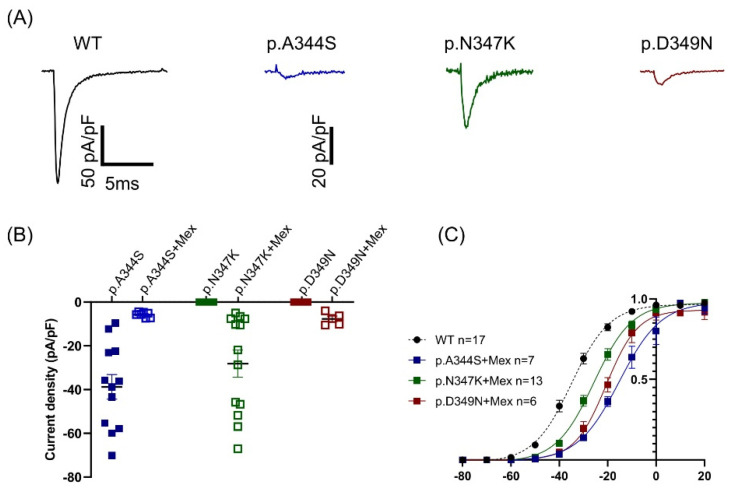
Effect of mexiletine on the mutated constructs. (**A**) Representative current traces of mutated channels recorded after mexiletine incubation in response to a depolarizing stimulus at −20 mV in HEK293 cells expressing the constructs as indicated: WT in black (for reference), p.A344S in blue, p.N347K in green, and p.D349N in red. (**B**). Scattered plot showing the distribution of the current density values obtained in absence (filled symbols) and in presence (empty symbols) of mexiletine. The black line represents the average values (see Table 1). (**C**) Fitting the data with the Boltzmann equation (see the Section 4) provided midpoint activation potential, as reported in Table 1. Data are shown as mean ± SEM.

Since an increasing amount of evidence has suggested that the functional ion channel current density can be pharmacologically modified, the potential rescue effects of the class Ib antiarrhythmic drug on cellular currents were explored [13], and HEK293 cells overexpressing mutated channels were subjected to an overnight incubation with 0.1mM mexiletine (10-fold higher than the maximal clinical concentration). The experimental findings revealed that the p.A344S channel was negatively influenced by mexiletine treatment, showing an 85% current reduction. However, the p.N347K mutation displayed a notably and pronounced rescue response (21%), while the p.D349N showed a modest, but still significant, 5% recovery effect (Figure 4A,B and Table 1). Moreover, the activation curve was shifted rightwards, although to a different extent, in all three mutations (Figure 4C, Table 1).

### 2.4. Structural Analysis

**Figure 5:**
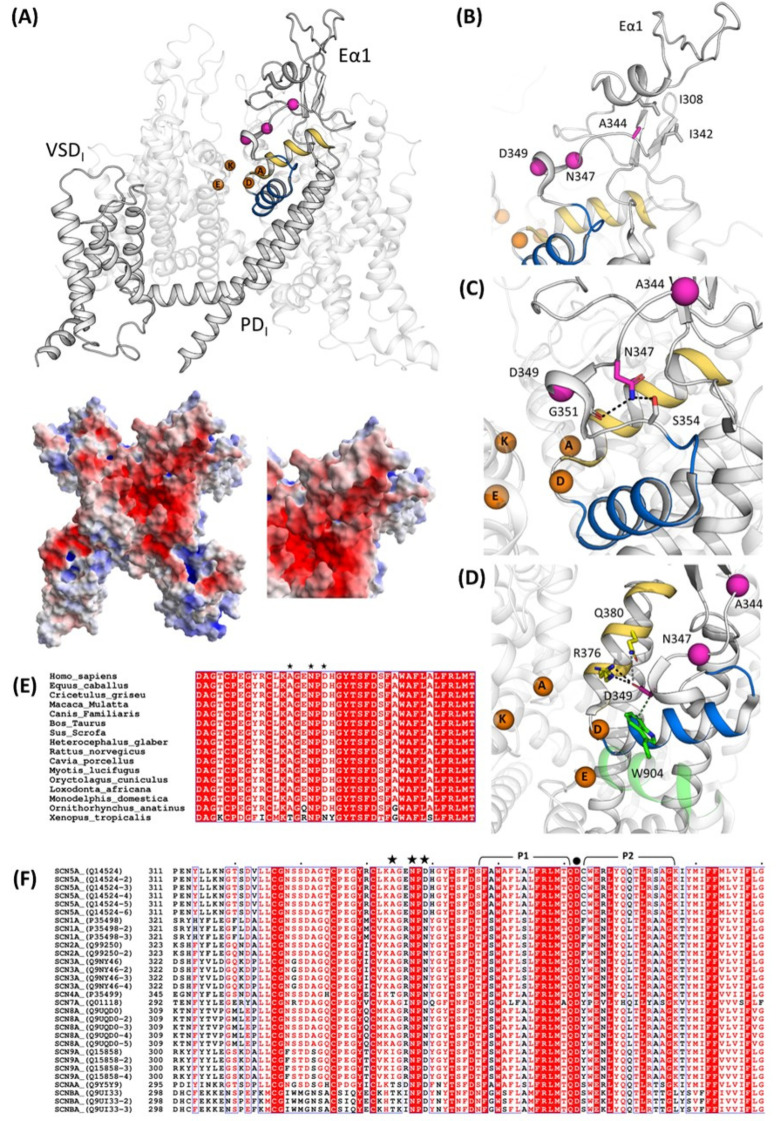
Structural analysis and mapping of the Nav1.5 mutations. (**A**) At the top, structural mapping of the Nav1.5 mutations reported in this study based on their cryo-EM structure (PDB id: 7DTC); on the bottom, a left extracellular view of Nav1.5 electrostatic potentials calculated with the APBS method and close-up of Eα1 on the bottom right. Interactions between p.A344 (**B**), p.N347, (**C**) and p.D349 (**D**) in WT Nav1.5. The Cα atoms of these residues are shown in magenta. The SF-supporting helices P1 and P2 are depicted in blue and yellow, respectively. The DEKA residues are shown as orange spheres. (**E**) Multiple sequence alignment of *SCN5A* genes in *Eukaryota* and (**F**) multiple sequence alignment of human SCN genes with their isoforms. Black stars indicate the mutation sites presented in this study. The selectivity filter (SF)-supporting pore helices P1 and P2 regions are lined above the sequence in black, and the residue p.D372, belonging to the DEKA motif (SF), is marked with a black dot.

The identification of three de novo *SCN5A* mutations in this study raises questions regarding the structural significance of these residues in Nav1.5. Their localizations and interactions have been investigated using the two available structures (PDB id: 6LQA and 7DTC) [14,15]. These mutations are localized in Nav1.5 repeat I, being part of the extracellular loop (Eα1) on top of the pore domain (PD-I), immediately above the selectivity filter (SF) and relative supporting pore helices P1 and P2 (Figure 5A). All the known mutations mapped to extracellular loops above the PD have been previously classified as “structural mutations”, capable of impairing protein folding and structural stability [15,16]. Similarly, the mutations investigated here can fall in the same classification. The role of positions 344, 347, and 349 in Nav1.5 is further underlined via the conservation across *Eukaryota*, (Figure 5E) as well as in most of the SCN genes (Figure 5F).

Among the three mutated amino acids, p.A344 is the most external and the farthest with respect to the SF. It interacts with the non-polar residues p.L308 and p.L342 (Figure 5B). In the case of p.A344S, the introduction of a slightly bulkier and polar residue may impact this otherwise hydrophobic pocket.

The second mutation site, p.N347, is deeper in the funnel-like region surrounding the extracellular portion of the SF. It forms hydrogen bonds with p.G351 and p.S354 (Figure 5C). The Nav1.5 mutants p.G351D and p.G351V have already been correlated with BrS, the former resulting in a loss-of-function of the channel [8,17]. After careful investigation of the surroundings of p.N347, the mutation p.N347K very likely triggers a local rearrangement to accommodate the lysine side chain, which is bulkier, more flexible, and positively charged, potentially resulting in an impairment to the correct orientation of the P1-SF-P2 motif. Furthermore, p.N347 is localized in a negatively charged area (Figure 5A), and such a negative patch may be perturbed by the introduction of a positively charged sidechain. Interestingly, the full conservation of a N in position 347 in all human SCN genes, as well as in the Nav1.5 orthologues (Figure 5E,F), accounts for its important role.

Finally, the p.D349 mutation site is also located in the PD at 14 Å from residue p.D372, belonging to the DEKA motif. It interacts with the P2I helix through a salt bridge with pR376 (which is conserved in all Nav1.5 isoforms and in some other Nav channels, Figure 5E,F) and with p.W904 from P2II through hydrophobic interactions (conserved in all human Nav channels). p.D349’s rich network of interactions connects P2I and P2II and, therefore, concurs to correctly place two out of four elements of the DEKA motif, since the helices P1 and P2 are known to contribute to the stabilization of the SF [16]. As for p.N347, p.D349 is part of a negative surface area of Nav1.5 (Figure 5A) and, therefore, the substitution of an aspartate with an asparagine, with high probability, has an impact on this, locally decreasing the negativity of the cliff that goes from the extracellular site into the pore. p.Q380, in close proximity (4.4 Å) with p.D349 and the two side chains, may be part of the same cluster of residues. It is worth saying that, in human Nav sequences, we observed a coordinated residue variability in positions 349 and 380 (Figure 5F); distinct pairs of residues were found as D/Q or N/L.

## 3. Discussion

From a clinical viewpoint, in the context of BrS, the three families described in this work are heterogeneous and present several possibilities. Family 1 is quite large, and all the seven members harboring the c.1030G>T (p.A344S) mutation tested positive for ajmaline, revealing complete penetrance. Notably, the ajmaline challenge has not been performed yet/has still to be performed on one pediatric carrier (the son of the proband’s third-grade cousin).

Concerning the c.1041C>A (p.N347K) mutation, as already reported by our group [18], our data indicate that one BrS patient within the family does not carry the mutation. Given the maternal inheritance of the c.1041C>A mutation in the *SCN5A* gene, it is possible that this specific mutation is not the one and only cause of BrS in this family. Therefore, it can be speculated that BrS might be caused by a different mutation only in this family member (for example, via a de novo mutation) or that c.1041C>A acts as a genetic modifier, able to worsen the clinical presentation of Brugada in all carriers. At the moment, the *SCN5A* c.1041C>A mutation is the only mutation that has been found in this family, considering the panel of 37 genes described in the literature regarding BrS.

Regarding the c.1045G>A (p.D349N) mutation, as also reported previously [18], the family is small with a confirmed pattern of inheritance. Unfortunately, the son of the proband, positive for the presence of the mutation, is not keen to collaborate for further analysis.

It is therefore evident that the genotype–phenotype correlation among BrS patients is challenging, due to both its variability and clinical behavior. Another source of complexity is the attribution of pathogenicity, even in the case of the *SCN5A* variants, partially due to the oligogenic inheritance of BrS. Certainly, the genetic test can be beneficial for risk stratification among probands and family members, but the relevance of common variants cannot be underestimated. Thus, it is important to remark that not all the cases of BrS are caused by *SCN5A* mutations, and that the genetic incomplete penetrance does not completely rule out the relation between a mutation and the syndrome.

These clinical cases represented the opportunity to shed light on the biophysical and structural features of three novel mutations in Nav1.5 located very close to each other (positions p.344, p.347, and p.349). All of them resulted in a loss-of-function of the channel, in which the predominant effect is the reduction (p.A344S)/abolishment (p.N347K and p.D349N) of the I_Na_, without showing a dominant-negative effect. Other biophysical properties, such as the subtle shift (4–5 mV) in the activation or inactivation curves, and the slowing in the kinetics of the fast inactivation process, could be quantitatively considered as less relevant. Notably, the three mutations fall in a region of the channel that is part of the P loop of domain I. This region extends from the aminoacidic residue p.M273 to p.S357, but already in the range between p.R340 and p.D356 (twenty-one residues long), five more mutations have been described (p.R340Q [19], p.G351V [17], p.G351D [8], p.T353I [20,21], and p.D356N [22]), suggesting its relevant role in the protein’s function and a possible mutational hot spot. All but one (p.R340Q) resulted in a protein loss-of-function, that most of the time, once again, corresponded to a significant reduction/abolishment in the current density. This effect is not surprising, since these mutations have been related to the BrS phenotype. The reduction in the current density has often been attributed to a trafficking defect. However, this assumption is questionable in the case of the three novel mutations we have described, as confocal images indicated the presence of the Nav1.5 channel on the plasma membrane. Despite the fact that it is important to recall the interpretive limitations associated with an image acquired through confocal microscopy (the analysis, in fact, remains only semi-quantitative, and even though the channel is visible in the plasma membrane, the possibility that the level of expression might be significantly diminished compared to the wild-type channel cannot be disregarded), such a result allowed for a deeper consideration in terms of the mechanism underlying the observed loss-of-function. In fact, p.A344S has the mildest effect on the Na^+^ channel’s current; a behavior similar to the one published for p.C335R and p.P336L [19,23], which precede it in sequence. On the other hand, for both p.N347K and p.D349N, probably due to their deeper positions and their interactions, the effect is more profound, as also suggested by the score assigned via the in silico prediction. In fact, in these two constructs, the inward current was completely absent, as it was described for the closely located p.G351-, p.T353-, and p.D356-mutated residues [8,17,20,21,22]. Indeed, the structural analysis suggested that the position p.A344, being more external, is quite far from the selectivity filter, while p.N347 is located deeper in the funnel-like region of the channel, and the introduction of a positive charge (K) may impair the correct orientation of the selectivity filter and of the two helices supporting the pore (the P1-SF-P2 motif). Finally, the p.D349 residue is in very close proximity to the selectivity filter, and, with its network of interactions, concurs to correctly place two out of four elements of the DEKA filter. Moreover, again, being part of a negative area, its substitution with a residue with an uncharged polar side chain (from D to N) may affect such a network and account for the dramatic effect. Thus, in general, and specifically for the p.A344S mutation, it cannot be excluded that the reduction in the current density may be explained via a decreased expression level at the plasma membrane (although no data are available at the moment); for the other two mutations, the destabilization of the selectivity filter seems to be one more aspect to be considered among the causes of the abolishment of the sodium current, despite the presence of the channel at the membrane.

Another interesting issue is related to the role of mexiletine in rescuing channel function. Mexiletine, classified as a class Ib antiarrhythmic drug, has been recognized for its binding to Nav1.5 channels at a local anesthetic-binding site within the fourth homologous domain’s sixth transmembrane segment. Beyond its use-dependent sodium channel blocking capability, it is also considered a pharmacological chaperone. Notably, mexiletine exhibits a certain level of tonic block, gauged as a first-pulse block, before close-state inactivation [24]. This implies an inherent affinity of mexiletine for the local anesthetic receptor site and, more broadly, for Nav1.5 channels, that may account for its escort activity. In this view, mexiletine’s contribution to the recovery of the I_Na_ is related to its capacity for inducing a conformational shift in misfolded channels that will appear normal to the quality control mechanism, thus permitting export from the endoplasmic reticulum (ER) and restoring membrane trafficking. The same impact on trafficking has not only been proposed for just Nav1.5 channels, but also to the hHERG channel and its blockers, which hinder anterograde channel delivery or facilitate rescue surface expression [25,26]. Regarding mexiletine’s application, a previous paper reported concentrations ranging from 10 µM (within clinical levels) to 500 µM that have been employed for 24–48 h of in vitro incubation. While not all mutations respond positively to this drug [27], those that do are located in different portions within the channel. They may be situated within a transmembrane helix, P-loop, linker between domains, or the C-terminus of the protein [13,28,29]. Notably, peak current recovery might coincide with significant shifts towards more negative potentials for both activation and inactivation voltage dependencies [13]. In contrast, for the first time, the p.A344S mutation demonstrated an 85% mexiletine-dependent peak current reduction, possibly linked to a 20 mV rightward shift in the activation curve. In contrast, the mutations p.N347K and p.D349N showcased the expected current recovery, similar to the effects seen with p.V1378M and p.G1743R, which are located in comparable positions between S5 and S6 of the third and fourth domains, but, again, with a positive shift in the gating properties [27,30]. It is also remarkable that p.N347K and p.D349N Nav1.5 were present on the cell membrane, despite the absence of an inward current, indicating a dysfunctional rather than completely impaired trafficking channel. A plausible rationale for the mexiletine-driven rescue is its potential to stabilize the selectivity filter’s position within the protein and counteract mutation-induced conformational alterations. However, translating this pharmacological in vitro rescue into a mutation-specific pharmacotherapy necessitates further exploration. Yet, the activation curve shift resulting from prolonged mexiletine exposure may pose risks of window current alterations, potentially affecting the action potential’s repolarization phase. Thus, paraphrasing the results obtained in this study, by treating the mutated channels for a considered chronic duration, the therapeutic intervention should be led by the consideration of which effect is prevalent, and the question that the clinician would be able to answer is: is the current density rescued by mexiletine enough to sustain phase 0 of the action potential, and thus the action potential upstroke velocity, but still not enough to alter the window current (that typically involves a minor fraction of the non-inactivated Nav1.5 channels)? In other words, is it possible to establish a threshold for the safety and efficacy of certain drugs among BrS patients in correlation with the presence of *SCN5A* mutations? The answer may avoid potentially harmful consequences [28].

### Limitations of the Study

While the presented study has shed light on the role of *SCN5A* mutations in BrS, we acknowledge limitations stemming from a relatively small patient cohort. Expanding genetic sequencing to encompass a broader range of genes and a more diverse BrS patient population is crucial. This approach promises deeper insights into the clinical significance of *SCN5A* mutations, potential genotype–phenotype correlations, and a more comprehensive understanding of BrS genetics. Due to BrS’s genetic complexity, we recognize the need for a broader multi-genomic exploration to uncover additional gene mutations or disease-associated factors. This comprehensive approach is essential for a more holistic understanding of BrS pathogenesis.

Moreover, our current research primarily relies on cellular and molecular studies. The incorporation of BrS animal models or induced pluripotent stem cell (iPSC)-derived cardiomyocytes in these kind of studies would enhance the validation of the impacts of specific mutations or therapeutic interventions within a more intricate biological system. These models can provide valuable in vivo and functional data, bridging the gap between genetic findings and their clinical implications.

Finally, in terms of deciphering the functional implications of mutations at the atomic level, experimental techniques, like X-ray crystallography and cryo-electron microscopy (cryo-EM), primarily provide the atomic coordinates of a protein’s structure. Of course, each approach has its own strengths and weaknesses: X-ray crystallography provides high-resolution structures, but requires protein crystallization, which can be difficult for some samples; cryo-EM offers high-resolution imaging and versatility, but the preparation of samples can be technically challenging, and there is a risk of generating structural artifacts or sample damage during the process. Electrostatic potential calculations, in spite of being limited by approximations, are computationally efficient and relatively fast; they provide enough information, and are cost-effective compared to our experimental methods; thus, they are useful for initial structural assessments and screening.

## 4. Materials and Methods

### 4.1. Subjects

Patients and relatives were evaluated in our Department of Arrhythmology and Electrophysiology at the hospital Policlinico San Donato. This study was conducted in accordance with the Declaration of Helsinki and written informed consent of human subjects was obtained for their participation in the study and for publication. The procedures employed were reviewed and approved by the local Ethics Committee (approver number: M-EC-006/A, rev. 1 March 2013).

### 4.2. DNA Extraction and Genetic Analysis

Genomic DNA was extracted from the peripheral blood of the probands and relatives using the Maxwell 16 Blood DNA Purification kit (Promega Italia, Milan, Italy). Their quality and concentration were determined using Nanodrop and Qbit (Thermo Fisher Scientific Italia, Monza, Italy). The samples were enriched using the Tru Sight One Sequencing kit (Clinical exome, Illumina, San Diego, CA, USA) and sequenced on the NextSeq500 platform (Illumina). The sequences were analyzed according to GATK Best Practice criteria, exploiting pipelines based on BWA, the Smith–Waterman algorithm, freebayes, SnpSift-SnpEFF, and BaseSpace Onsite. The next-generation sequencing (NGS) panel was only used for the three probands of each family, while all other family members were studied with Sanger sequencing. This panel contained 37 genes that have been described in the BrS research literature (*ABCC9*, *ACTC1*, *AKAP9*, *CACNA1C*, *CACNA2D1*, *CACNB2*, *DSC2*, *DSG2*, *DSP*, *FLNC*, *GPD1L*, *HCN4*, *JUP*, *KCND2*, *KCND3*, *KCNE1L*, *KCNE3*, *KCNE5*, *KCNH2*, *KCNJ8*, *MOG1*/*RANGRF*, *MYBPC3*, *MYH7*, *MYL2*, *MYL3*, *PKP2*, *PLN*, *SCN10A*, *SCN1B*, *SCN2B*, *SCN3B*, *SCN5A*, *SEMA3A*, *TNNI3*, *TNNT2*, *TPM1*, and *TRPM4*). The mean coverage of the sequenced regions was 123×. The analyzed regions covering <20× include the regions 91708393–91708423 (chromosome 7, gene *AKAP9*, transcript NM_005751), 2791187–2191220 (chromosome 12, gene *CACNA1C*, transcript NM_199460), and 150644885–150644904 (chromosome 7, gene *KCNH2*, transcript NM_000238).

### 4.3. Plasmid Generation

Wild-type (WT) *SCN5A* complementary DNA (cDNA) was subcloned into the pcDNA3.1 plasmid, with a tandem twin Strep tag and FLAG tag at the N terminus of Nav1.5. The mutations were engineered via site-directed mutagenesis through the QuikChange II site-directed mutagenesis kit (Agilent Technologies Italia, Milan, Italy), according to the manufacturer’s instructions. The oligonucleotides used for the three mutations were: c.1030 G>T, forward 5′-GCTACCGGTGCCTAAAGTCAGGCGAGAACC-3′, reverse 5′-GGTTCTCGCCTGACTTTAGGCACCGGTAGC-3′; c.1041 C>A, forward 5′-AAAGGCAGGCGAGAAACCCGACCACGGCTAC-3′, reverse 5′-GTAGCCGTGGTCGGGTTTCTCGCCTGCCTTT-3′; and c.1045 G>A, forward 5′- CAGGCGAGAACCCCAACCACGGCTACACC-3′, reverse 5′-GGTGTAGCCGTGGTTGGGGTTCTCGCCTG-3′. Sanger sequencing was then performed to confirm the accurate incorporation of the mutation and validate the sequence’s integrity. The *SCN1B* gene, which encodes for the human cardiac β1 subunit, was inserted into a pIRES vector that was engineered to contain EGFP as a reporter gene (a kind gift from Prof. Hugues Abriel, University of Bern, Bern, Switzerland).

### 4.4. Cell Culture and Transfection

The HEK293 (human embryonic kidney 293) cell line was obtained from ATCC. Cells were cultured in a controlled environment (5% CO_2_, 37 °C) and maintained in an appropriate medium (DMEM/F12; Gibco-Thermo Fisher Scientific Italia, Monza, Italy) supplemented with 10% fetal bovine serum (FBS, FBS; Sigma-Aldrich—Merk Life Science, Milan, Italy), 2 mM L-Glutamine, 100 U/mL, and 100 μg/mL Pen/Strep (Euroclone, Pero, Italy). Equal amounts of *SCN5A* (0.5 µg, WT or mutant channel) and *SCN1B* subunits cDNA (1:1 ratio) were transiently transfected using the jetPRIME reagent (PolyPlus transfection, Euroclone, Pero, Italy), according to the manufacturer’s instructions. In order to mimic the genetic balance of affected individuals, 0.25 µg of the WT and 0.25 µg of the variant alpha subunit-encoding plasmid, as well as 0.5 µg of the beta 1 subunit, were co-transfected (0.5:0.5:1 ratio). Following this step, 48 h after transfection, the cells were dispersed using a trypsin/EDTA solution (Gibco-Thermo Fisher Scientific Italia, Monza, Italy), gently centrifuged at room temperature (RT) at a speed of 200× *g* for 5 min, and resuspended. For experiments focused on the rescue of the I_Na_, transfected cells were incubated overnight with mexiletine (mexiletine hydrochloride, Sigma-Aldrich - Merk Life Science, Milan, Italy ) at a concentration of 0.1 mM [20].

### 4.5. Functional Analysis

#### 4.5.1. Automated Patch Clamp

Automated patch clamp recordings were performed at room temperature (RT) using the Nanion Patchliner platform (Nanion Technologies, Munich, Germany). The transfected cells, after dispersion in trypsin and centrifugation, were then resuspended in an external solution at a final concentration of 500,000 cells/mL. The cells were allowed to recover at 4 °C for 20 min before proceeding with the automated recordings. According to company recommendations, single-hole medium resistance (1.8–3 MΩ) NPC-16 chips were used to record the Na^+^ current. Pulse generation and data collection were performed using PatchControl v3.01.19 (Nanion Technologies, Munich, Germany ) and PatchMaster v2X92 (Heka, Multi Channel Systems MCS GmbH, Reutlingen, Germany) software. Whole-cell currents were filtered at 10 KHz, the sampling rate was set to 20 µs, and series resistances were automatically compensated. The external recording solution contained (mM): 80 NaCl, 60 NMDG, 4 KCl, 2 CaCl_2_, 1 MgCl_2_, 5 D-Glucose monohydrate, and 10 Hepes, (pH 7.4 with HCl, osmolarity adjusted to 289 mOsm/Kg). The internal solution was composed of the following (mM): 110 CsF, 10 NaCl, 10 CsCl, 10 Hepes, and 10 EGTA (pH 7.2 with CsOH, osmolarity > 280 mOsm/kg). To achieve high-resistance seals, a seal enhancer solution was added, containing (mM): 80 NaCl, 60 NMDG, 4 KCl, 10 CaCl_2_, 1 MgCl_2_, 5 D-Glucose monohydrate, and 10 Hepes, (pH 7.4 with HCl, osmolarity adjusted to 313 mOsm/Kg). The holding potential was −120 mV. Steady-state activation was studied pulsing from −80 mV to +20 mV (+5 mV increments, 25 ms duration), while the steady-state inactivation protocol had an initial 500 ms duration step (from −140 mV to +10 mV, increments of +10 mV), followed by a −10 mV test pulse (20 ms durations). Steady-state activation and availability curves were fitted with a Boltzmann function: y = 1/(1 + exp((V − V_1/2_)/k)), where y is the relative current, V is the membrane potential, V_1/2_ is the half-maximal voltage, and k is the slope factor. The onset of the fast inactivation process was studied, fitting the decay of the peak current traces elicited by a 25 ms depolarizing pulse from −120 to +20 mV with a bi-exponential function (the data in Table 1 only show the value of the τ fast @ −20 mV).

#### 4.5.2. Manual Patch Clamp

After dispersion with trypsin and centrifugation, the cells were plated onto 35 mm plastic Petri dishes and used for whole-cell patch clamp experiments a few hours later to allow attachment. Voltage clamp experiments were conducted at RT, and only GFP-expressing (green) cells were selected for recording. The internal and external solutions used for the recordings were the same solutions that were adopted for automated patch clamp analysis. Borosilicate glass patch pipettes (2–3 MΩ) were pulled with a P-1000 Flaming-Brown micropipette puller (Sutter Instrument). Series resistance (Rs) were compensated, and the compensation was readjusted before each protocol. Recovery from inactivation was studied through a two-step protocol at −10 mV (500 ms and 20 ms duration, respectively), separated by a −120 mV pulse of increasing duration from 0.1 ms to 1 s. Also, the time dependence of the onset of intermediate and slow inactivation was measured with a two-pulse protocol: the first pulse, P1, stepped from the holding potential of −120 mV to −20 mV (increasing duration from 1 to 1000 ms), followed by a step back to −120 mV for 20 ms to let the channels recover from the fast inactivation, and the second pulse, P2, to −20 mV (duration 20 ms) enables the recording of the sodium current. The resulting P2/P1 ratio was normalized and plotted against the P1 duration [31,32]. Data were acquired with a Multiclamp 700B amplifier and Digidata 1550B and pClamp 10.3 software, and analyzed with Clampfit 10.7 software (all Axon Instruments, Molecular Devices, San Jose, CA, USA).

#### 4.5.3. Statistical Analysis

Functional data are presented as the mean ± SEM of at least three independent experiments; *n* denotes the number of cells. A two-way ANOVA was performed for multiple comparisons, followed by a modified *t*-test with Fisher’s correction (OriginPro 8; OriginLab Corporation, Northampton, MA, USA ). Values of *p* < 0.05 were considered significant, and indicated with * or ^#^ in the figures and table.

### 4.6. Immunofluorescence Staining

For the immunofluorescence staining experiments, the SCN1B gene was subcloned in a pCDNA3.1 vector devoid of the GFP reporter gene in order to prevent the overlapping fluorescence of the GFP and the secondary antibody used. HEK293 cells were plated on a glass slide coated with gelatin 0.1% in PBS; following this step, 48 h after transfection, they were fixed using PFA 4% in PBS for 15 min at RT. Three washes of 5 min each at RT in PBS high salt buffer (PBS-HS) were performed. Before antibody staining, the cells were incubated with the permeabilization solution (gelatin 0.4% Triton X-100 0.6%, phosphate buffer 40 mM, NaCl 0.9 M, and saponin 0.1%) for 15 min at RT. Two washes were then conducted with the PBS-HS solution. The non-specific binding sites of the primary antibody were blocked with PBS + 0.5% bovine serum albumin (BSA) for 1 h at RT. The excess was removed with two 5 min washes in PBS-HS. The cells were incubated with permeabilization buffer (20 mM PBS pH 6.8, 2% gelatin, 0.1% Triton X-100 solution, 0.45 mM NaCl, 1% albumin, and 0.1% saponin) for 15 min at RT. The primary (Anti.Nav1.5 Rabbit monoclonal antibody, Cell Signaling Europe, Leiden The Netherlands, Catalogue # 14421, diluted 1:400) and secondary (Donkey Anti-rabbit Alexa Fluor 488, Thermo Fisher Scientific Italia, Monza, Italy, Catalogue # A-21206, diluted 1:600) antibodies were incubated overnight (o/n) at 4 °C, and for 1 h at RT in the dark, respectively, in permeabilization solution. The excess antibodies were then removed with two washes (5 min, RT) in PBS-HS and two washes in PBS low salt (LS). The slides were coverslipped with DAKO fluorescence mounting medium. The Zeiss LSM710 confocal microscope, equipped with a 63× oil immersion objective, was used for image acquisition as a single optical section. Zeiss ZEN Microscope Lite software version and ImageJ 1.48V were used for image processing.

### 4.7. Structural Analysis

Nav1.5 structural data were retrieved from wwPDB (PDB ID: 7DTC, 6LQA). Electrostatic potential calculations and figures were generated using PyMOL (Schrödinger, LLC, Munich, Germany. The PyMOL Molecular Graphics System, Version 2.5.6). The electrostatic potential was calculated using the Adaptive Poisson–Boltzmann Solver (APBS) method [33]. The sequences of human SCN genes (UniProt accession No. P35498, P35499, Q01118, Q14524, Q15858, Q99250, Q9NY46, Q9UI33, Q9UQD0, and Q9Y5Y9) were retrieved from UniProt and aligned in MEGA 11 [34] using the MUSCLE algorithm [35]. The multiple sequence alignment presented (Figure 5) was generated with ESPript [36].

## 5. Conclusions

In conclusion, our data confirm that the clinical management of patients affected by Brugada syndrome today requires a multidisciplinary approach. The genetic test is quite valuable, and should be used in conjunction with both familial and clinical evaluations to aid stratification. Certainly, many common variants might contribute to the BrS clinical phenotype, as published by Tadros and coworkers [37]. Therefore, the combination of in silico prediction (a precious tool that helps in prioritizing mutations for further investigation) and structural modeling, together with functional tests, constitute a robust approach to contribute to a disease phenotype characterization, especially when considering the oligogenic model of BrS. Finally, drug testing is crucial for personalized treatment, but should consider the effects on mutated channels. Establishing safety and efficacy thresholds for drugs in correlation with *SCN5A* mutations can prevent harmful consequences.

## Figures and Tables

**Table 1:** Electrophysiological parameters of the three Nav1.5-mutated constructs with respect to the WT channel, in absence and in presence of 0.1 mM mexiletine. Data are shown as mean ± SEM.

**(A)**							
		**WT**	**p.A344S/WT**	**p.A344S**	**p.N347K/WT**	**p.D349N/WT**	
***n***		**17**	**20**	**12**	**27**	**24**	
Current density @ −20 mV	(pA/pF)	−134.5 ± 14	−77.8 ± 9 *	−38.8 ± 6 *^,#^	−63.3 ± 7 *	−69.5 ± 8 *	
Activation	V_1/2_ (mV)	−33.7 ± 1	−29.3 ± 5 *	−29.2 ± 5 *	−29.7 ± 1*	−31.1 ± 1	
	k	7.8 ± 0.5	8.9 ± 0.3	8.3 ± 0.5	8.4 ± 0.3	7.8 ± 0.6	
Inactivation	V_1/2_ (mV)	−82.5 ± 2	−82.8 ± 2	−77.5 ± 1 *	−83.2 ± 2	−77.6 ± 1 *	
	k	5.4 ± 0.3	6.5 ± 0.3	5.3 ± 0.3	6.1 ± 0.3	5.2 ± 0.2	
Fast inactivation @ −20 mV	$\tau \;\left(\mathrm{ms}\right)$	0.74 ± 0.06	0.85 ± 0.09	1.03 ± 0.09 *	0.95 ± 0.08	0.74 ± 0.04	
*n*		12	7	4	23	17	
Recovery from inactivation	$\tau 1\;\left(\mathrm{ms}\right)$	8.8 ± 0.6	10.6 ± 4	7.8 ± 0.8	7.4 ± 0.7	8.3 ± 0.7	
	$\tau 2\;\left(\mathrm{ms}\right)$	210.4 ± 26	450.5 ± 191	396.7 ± 187	171.9 ± 32	304.4 ± 44	
**(B)**							
**+Mexiletine 0.1 mM**							
		**p.A344S**	**Change**	**p.N347K**	**Change**	**p.D349N**	**Change**
Current density @ −20 mV	(pA/pF)	−5.72 ± 0.4	−85% *	−28.15 ± 6	+21% *	−7.45 ± 1	+5% *
Activation	V_1/2_ (mV)	−14.5 ± 0.5	+19 ^#^	−24.7 ± 0.5	+9 ^#^	−19.1 ± 0.8	+14.6 ^#^
	k	8.9 ± 0.5		8.1 ± 0.4		7.8 ± 0.7	
*n*		7		13		6	

(**A**) *n* indicates the number of cells tested from a minimum of three different experiments, * and ^#^ indicate *p* < 0.05 vs. WT and p.A344S/WT, respectively; (**B**) *n* indicates the number of cells tested from a minimum of three different experiments, * indicates *p* < 0.05 vs. the same parameter studied in absence of 0.1 mM mexiletine, and ^#^ indicates the shift amplitude vs. the WT recorded in absence of 0.1 mM mexiletine.

## Data Availability

The raw data supporting the conclusions of this manuscript will be made available by the authors, without undue reservation, to any qualified researcher.

## Supplementary Materials

The following supporting information can be downloaded at: https://www.mdpi.com/article/10.3390/ijms242015089/s1.
