# Supplementary material for: Unravelling Novel SCN5A Mutations Linked to Brugada Syndrome: Functional, Structural, and Genetic Insights
Source: Int J Mol Sci. 2023 Oct 11;24(20):15089. doi: 10.3390/ijms242015089 (PMC10606416; doi:10.3390/ijms242015089)
Supplement: Supplementary file 1 [file ijms-24-15089-s001.zip › ijms-2628639-supplementary.pdf]

Supplementary Material

Assessment of Family Members

Family 1.

The proband's 56-year-old asymptomatic mother tested positive for ajmaline. However, the EPS did not induce sustained ventricular fibrillation (VF). Genetic testing revealed that she is a heterozygous carrier of the c.1030G>T mutation in a single copy of the *SCN5A* gene, although the inheritance pattern remains unknown.

The proband 31-year-old asymptomatic sister underwent ajmaline testing, which yielded positive results, as well as the induction of sustained ventricular fibrillation during EPS. Subsequently, an ICD was implanted. Genetic testing confirmed the presence of the heter-ozygous c.1030G>T mutation, in a single copy of the *SCN5A* gene, inherited maternally.

The proband's 25-year-old asymptomatic brother, who tested positive for ajmaline, did not exhibit sustained ventricular fibrillation during EPS. Genetic testing revealed that the brother also carries the heterozygous c.1030G>T mutation in a single copy of the *SCN5A* gene, inherited maternally.

The proband's 18-year-old asymptomatic brother tested positive for ajmaline and genetic testing confirmed his status as a heterozygous carrier of the familial c.1030G>T mutation in a single copy of the *SCN5A* gene, with maternal inheritance.

The proband's 51-year-old maternal uncle occasionally exhibited a spontaneous type 1 BrS ECG pattern. Ajmaline testing confirmed positivity, and he displayed inducibility for sustained ventricular fibrillation during EPS. Consequently, an ICD was implanted. Genetic testing indicated that he carries the heterozygous c.1030G>T mutation in a single copy of the *SCN5A* gene, with an unknown inheritance pattern.

The proband's 40-year-old third-grade cousin had a family history of sudden death during fever. Notably, her 72-year-old father experienced sudden death while being treated with a common antibiotic for bronchitis. Ajmaline testing resulted positive for type 1 BrS ECG, whereas no ventricular arrhythmias were inducible at the EPS. Genetic testing con-firmed her status as a heterozygous carrier of the familial c.1030G>T mutation in a single copy of the *SCN5A* gene, although the inheritance pattern remains unconfirmed.

One of the two children of the proband's third-grade cousin also carries the c.1030G>T mutation but is currently in the pediatric age group and ajmaline challenge has not been performed yet. Consequently, the parents were advised to perform an ECG during fever episodes to assess the presence of a type 1 Brugada pattern and potentially unveil the phenotypic expression associated with the familial variant.

Family 2.

The proband's 54-year-old sister presented with a personal history of recurrent palpitations. Sodium channel blocker (SCB) test with flecainide was performed at another center, which showed a type 1 BrS ECG inducibility. The EPS demonstrated the induction of sustained VF, therefore she received an ICD. Genetic testing confirmed her status as a heterozygous carrier of the c.1041C>A mutation in a single copy of the *SCN5A* gene, alt-hough the inheritance pattern remains unknown.

Two sons of the proband died suddenly during sleep at the age of 23 and 22. Their genetic status is unknown.

The proband's 35-year-old daughter experienced episodes of syncope during puberty and again during the eighth week of her first pregnancy. Therefore, at that time due to her condition, she couldn’t undergo ajmaline challenge. The genetic testing (Sanger sequencing on peripheral blood) revealed her heterozygous carrier status for the familial c.1041C>A mutation in a single copy of the *SCN5A* gene, with maternal inheritance. Following delivery, she underwent SCB test with ajmaline, which showed conversion to type 1 BrS ECG pattern.

The proband's 27-year-old asymptomatic son underwent ajmaline testing, which yielded positive results. Surprisingly, he was the only family member who remained completely asymptomatic and did not

exhibit right bundle branch block on both basal and modified EKG. Genetic testing revealed that he did not carry the familial c.1041C>A mutation in a single copy of the *SCN5A* gene.

Family 3.

The proband's 65-year-old mother had a personal history of atrial fibrillation. Due to her family history, she underwent a flecainide challenge test at another hospital, which showed type 1 ECG pattern inducibility. The EPS demonstrated the induction of ventricular fibrillation. Consequently, an ICD was implanted. Genetic testing confirmed her status as a heterozygous carrier of the c.1045G>A mutation in a single copy of the *SCN5A* gene, although the inheritance pattern remains unknown.

The proband's 41-year-old sister experienced lipothymia during puberty and received a clinical diagnosis of mitral valve prolapse without significant mitral regurgitation. The ECG was not suspicious for BrS pattern and ajmaline challenge resulted negative. Subse-quently, genetic testing (Sanger sequencing on peripheral blood) indicated that she is not a heterozygous carrier of the familial mutation.

The proband's son is a male child who was at primary school at the time of first genetic counselling. He harbors the same familial c.1045G>A mutation in a single copy of *SCN5A* gene with paternal inheritance; however, the parents refused any further medical examination.

Genetic Testing Results and In Silico Prediction.

c.1030G>T
(https://varsome.com/variant/hg38/SCN5A%3Ac.1030G%3ET?annotation-mode=germline).
CADD: 24 (cut off of pathogenicity is >20), Polyphen2: 0.866 (possibly damaging), SIFT: deleterious. Mutation Taster: deleterious.
Freq European non-Finnish: 1/112774 alleles

c.1041C>A
(https://varsome.com/variant/hg38/SCN5A%3Ac.1041C%3EA?annotation-mode=germline).
CADD: 23.4 (cut off of pathogenicity is > 20), Polyphen2: 0.877 (possibly damaging), SIFT: deleterious. Mutation tester: deleterious.
Freq European non-Finnish: not available

c.1045G>A
(https://varsome.com/variant/hg38/SCN5A%3Ac.1041C%3EA?annotation-mode=germline).
CADD: 22.7 (cut off of pathogenicity is > 20), Polyphen2: 0.936 (probably damaging), SIFT: deleterious
Mutation tester: deleterious.
Freq European non-Finnish 1/128340 alleles

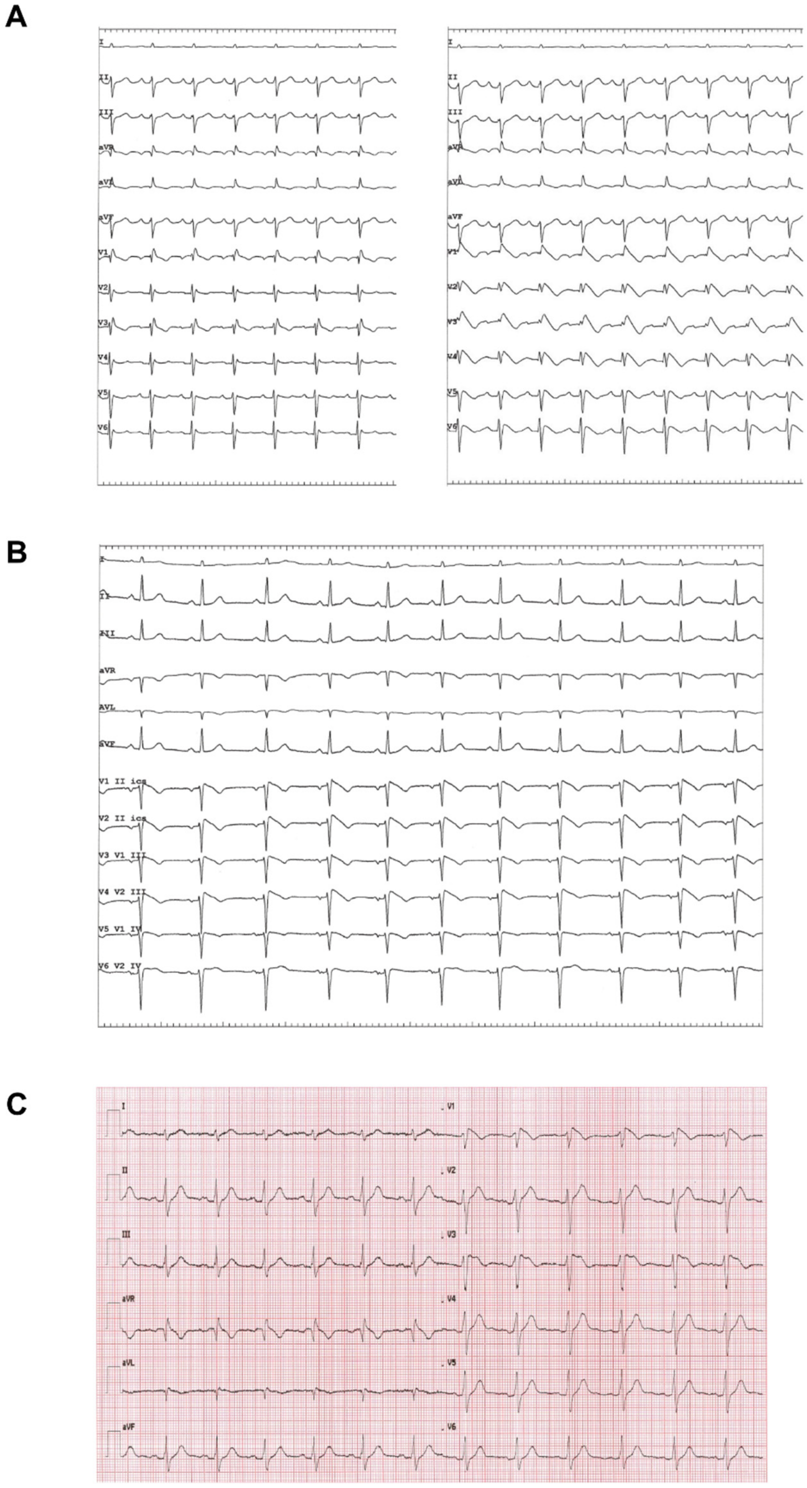

Figure S1. Complete ECG of the families' proband. (A) Basal (left) and ajmaline-induced (right) pattern of family 1 proband. (B) and (C) ECG of probands of familiy 2 and 3, respectively, showing spontanous Type-1 BrS pattern.
